# Longwing (*Heliconius*) butterflies combine a restricted set of pigmentary and structural coloration mechanisms

**DOI:** 10.1186/s12862-017-1073-1

**Published:** 2017-11-21

**Authors:** Bodo D. Wilts, Aidan J. M. Vey, Adriana D. Briscoe, Doekele G. Stavenga

**Affiliations:** 1 Computational Physics, Zernike Institute for Advanced Materials, University of Groningen, Nijenborgh 4, NL-9747AG Groningen, The Netherlands; 2 Adolphe Merkle Institute, University of Fribourg, Chemin des Verdiers 4, CH-1700 Fribourg, Switzerland; 3 Department of Ecology and Evolutionary Biology, University of California, Irvine, CA 92697 USA

**Keywords:** Light scattering, Signaling, Thin films, Nymphalidae, Ommochromes, Aposematism, Butterflies

## Abstract

**Background:**

Longwing butterflies, *Heliconius sp.*, also called heliconians, are striking examples of diversity and mimicry in butterflies. Heliconians feature strongly colored patterns on their wings, arising from wing scales colored by pigments and/or nanostructures, which serve as an aposematic signal.

**Results:**

Here, we investigate the coloration mechanisms among several species of *Heliconius* by applying scanning electron microscopy, (micro)spectrophotometry, and imaging scatterometry. We identify seven kinds of colored scales within *Heliconius* whose coloration is derived from pigments, nanostructures or both. In yellow-, orange- and red-colored wing patches, both cover and ground scales contain wavelength-selective absorbing pigments, 3-OH-kynurenine, xanthommatin and/or dihydroxanthommatin. In blue wing patches, the cover scales are blue either due to interference of light in the thin-film lower lamina (e.g., *H. doris*) or in the multilayered lamellae in the scale ridges (so-called ridge reflectors, e.g., *H. sara* and *H. erato*); the underlying ground scales are black. In the white wing patches, both cover and ground scales are blue due to their thin-film lower lamina, but because they are stacked upon each other and at the wing substrate, a faint bluish to white color results. Lastly, green wing patches (*H. doris)* have cover scales with blue-reflecting thin films and short-wavelength absorbing 3-OH-kynurenine, together causing a green color.

**Conclusions:**

The pigmentary and structural traits are discussed in relation to their phylogenetic distribution and the evolution of vision in this highly interesting clade of butterflies.

**Electronic supplementary material:**

The online version of this article (10.1186/s12862-017-1073-1) contains supplementary material, which is available to authorized users.

## Background

The colorful wings of butterflies have long caught the eye of natural biologists [1]. Butterfly wing coloration patterns have numerous functions, including mate attraction and identification, concealment, and warning (aposematic) signaling [2–4]. Because wing coloration often plays multiple functions simultaneously [5], a possible conflict exists between the expression of the different ways of signaling, which can be overcome by spatially or dynamically separating the color signals. For instance, the coloration of the ventral wing side is commonly involved in predator avoidance (e.g. by camouflage), since most butterflies fold their wings above the body when at rest, so that only the ventral wing sides are visible [5–7]. The often brightly-colored dorsal wing sides predominantly function in intra-specific communication, which however can also make the butterflies highly visible to predators. Aposematic species, displaying visible warning colors [2, 3], are often involved in complex mimicry ‘rings’, where multiple species converge on a similar coloration pattern [3, 4, 8, 9]. Such mimicry has clear fitness advantages, as the participating species all benefit from looking alike. However, in order to successfully reproduce, members of species involved in mimicry rings must be able to distinguish conspecifics from heterospecifics [10, 11].

The mechanisms underlying coloration are usually distinguished in having a physical or chemical basis. Physical colors are created by orderly arranged nanostructures, and chemical colors are due to wavelength-selective absorbing pigments. The various physical and chemical mechanisms contributing to butterfly wing coloration are often considered to operate separately from each other [12], but sometimes pigmentary and structural coloration combine non-trivially [13–15]. For example, in the wings of cabbage whites (pierids), the UV-absorbing pigment leucopterin, causing low wing reflectance in the ultraviolet, is located in densely packed, ellipsoidal-shaped beads in the wing scales that serve to enhance the scattering in the (for humans) visible wavelength range, resulting in brilliant white wings [14, 16, 17]. Birdwing butterflies (*Ornithoptera*) have extremely colorful wings due to specially-structured wing scales, which contain papiliochrome pigments acting as a spectral filter on a chirped multilayer reflector, thus tuning the scale color [15]. In the nymphaline peacock butterfly, *Aglais io*, the cover scales in the blue eyespots at the dorsal wings are blue due to the lower lamina acting as a blue-reflecting thin film [18]. The ground scales in the eyespots are black, because of highly concentrated melanin, thus serving as a contrast enhancing background. However, when the ground scales have the same structure as the blue cover scales, the stack of scales together with the wing substrate create a faint-bluish or even whitish color [18].

Here, we investigate the spectral properties of several *Heliconius* species in order to identify the main pigmentary and structural mechanisms that determine the wing coloration patterns. Heliconians present an attractive study system, because the coloration of many species is bright and simple-patterned. Furthermore, the genetic processes that determine the wing coloration and the role of color in the ecology of the species in this group have been well studied [2, 9, 10, 19, 20]. *Heliconius* species further present the prime example of aposematic coloration, rapid speciation and the presence of Müllerian mimicry rings [2, 21–23]. To elucidate the spectral properties of heliconian butterfly wing scales, we applied various optical methods as well as electron microscopy. We place the study in a phylogenetic context in order to demonstrate how some coloration mechanisms are restricted to certain taxa, while others are widespread. We highlight the value of using reflectance spectrometry to quantify and distinguish the spectral properties of butterfly wings.

## Methods

### Specimen

We investigated 12 *Heliconius* species (and a number of subspecies) (Additional file 1: Table S1) representing ~25% of species in the genus [24]. Specimens were either ordered from commercial suppliers (dried specimen were obtained from Tropical Butterflies and Insects of America, Tampa, FL, USA and Worldwide Butterflies, Dorset, UK; pupae of *H. doris* were obtained from Costa Rica Entomological Supply, Costa Rica) or were from the collection of the National Museum of Natural History *Naturalis*, Leiden, the Netherlands. Photographs of pinned specimens were taken with a Nikon D70 equipped with an F70 macro objective lens and a Nikon SB-800 ring flash.

### Scanning electron microscopy

The morphological structure of wing scales was visualized with a Tescan Mira 3 field-emission scanning electron microscope (SEM), using samples sputtered with a ~3 nm thick layer of  platinum/palladium (80:20 wt%).

### Imaging scatterometry

We applied imaging scatterometry to visualize the far-field angular distribution of the light scattered from single scales (2–5 scales per specimen), glued at the end of pulled glass micropipettes [25, 26]. The sample was positioned in the first focal point of the scatterometer’s ellipsoidal mirror, which collects light from a full hemisphere. A xenon lamp was used for illumination, producing a narrow aperture beam (5°) and a small spot size (diameter ~30 μm). A piece of magnesium oxide served as a white diffuse reference object. Scatterogram images were acquired by an Olympus DP70 camera and were subsequently corrected for geometrical distortions using a Matlab routine.

### Spectrometry

Reflectance spectra of intact wings (average of 2–5 individual spectra of all 12 investigated species) were measured with a bifurcated probe linked to a halogen/deuterium light source and an Avantes AvaSpec-2048-2 (Avantes, Apeldoorn, NL) CCD detector array spectrometer. The angle of illumination was about normal with respect to the wing surface. A microspectrophotometer (MSP), consisting of a Leitz Ortholux microscope and the Avantes spectrometer, was used to measure reflectance spectra from both sides of single scales (abwing – upper side; adwing – lower side), which were removed from the wing and glued to the tip of pulled glass pipettes. A white diffuse standard (Avantes WS-2) was used as a reference for all reflectance measurements. Absorbance spectra of single wing scales were obtained with the MSP by measuring the transmittance of single scales embedded in immersion fluid (Cargille Labs, Cedar Grove, NJ, USA; Series A) with a refractive index of 1.56, closely matching the refractive index of chitin [27].

### Pigment extraction and absorbance spectra

Pigments from the red, orange and yellow scales on the wings were extracted from a total of 1 individual per species in a solution of 50:1 methanol: 1 M hydrochloric acid. Wing fragments excised from colored patches were placed in 600 μl of this solution in Eppendorf tubes for ~1 h. Solutions were then centrifuged for 4 min at 14000 rpm (Eppendorf centrifuge 5418), and the supernatant removed for liquid spectrometry. A liquid UV-VIS spectrometer (Perkin Elmer LAMBDA 365) was used to measure the absorbance spectra of the pigment in solution against a reference of pure MeOH:HCl solution. All chemicals (MeOH and HCl) were purchased from Sigma Aldrich and used as received.

### Ancestral state reconstruction of wing pigmentary and structural coloration

The Bayesian phylogeny of Kozak et al. [24] including branch lengths was used to trace the evolution of two types of scales that derive their color primarily from pigmentation [*i*) 3-OHK (yellow), *ii*) xanthommatin/dihydroxanthommatin (orange/red)], three types of scale that derive their color solely from their structure [*iii*) simple blue scales (blue), *iv*) simple blue scales stacked (white), *v*) multi-ridge reflectors coloration (blue)], and one type of scale whose color derives from both [*vi*) simple blue scales containing 3-OHK (green)]. A total of sixteen *Heliconius* species/subspecies with representatives of all seven major clades and one outgroup species in the genus *Eueides* were included (Additional file 1: Table S1). Species entirely missing any particular trait were coded as 0, species with reflectance spectra characteristic of a certain trait were coded as 1. Ancestral state reconstruction was performed in Mesquite (ver. 3.2; ref. [28]) using the maximum likelihood option with default settings.

## Results

### Colors and reflectance spectra of longwing butterflies

The wings of *Heliconius* butterflies are well-known for their striking wing patterns that feature bright yellow, orange or red-colored patches in a velvet black frame. This frame may show metallic blue patches in some species. The butterflies have a total wingspan of 4–6 cm and the hindwings are much smaller than the forewings, resulting in a highly asymmetrical appearance characteristic for heliconians.

**Fig. 1:**
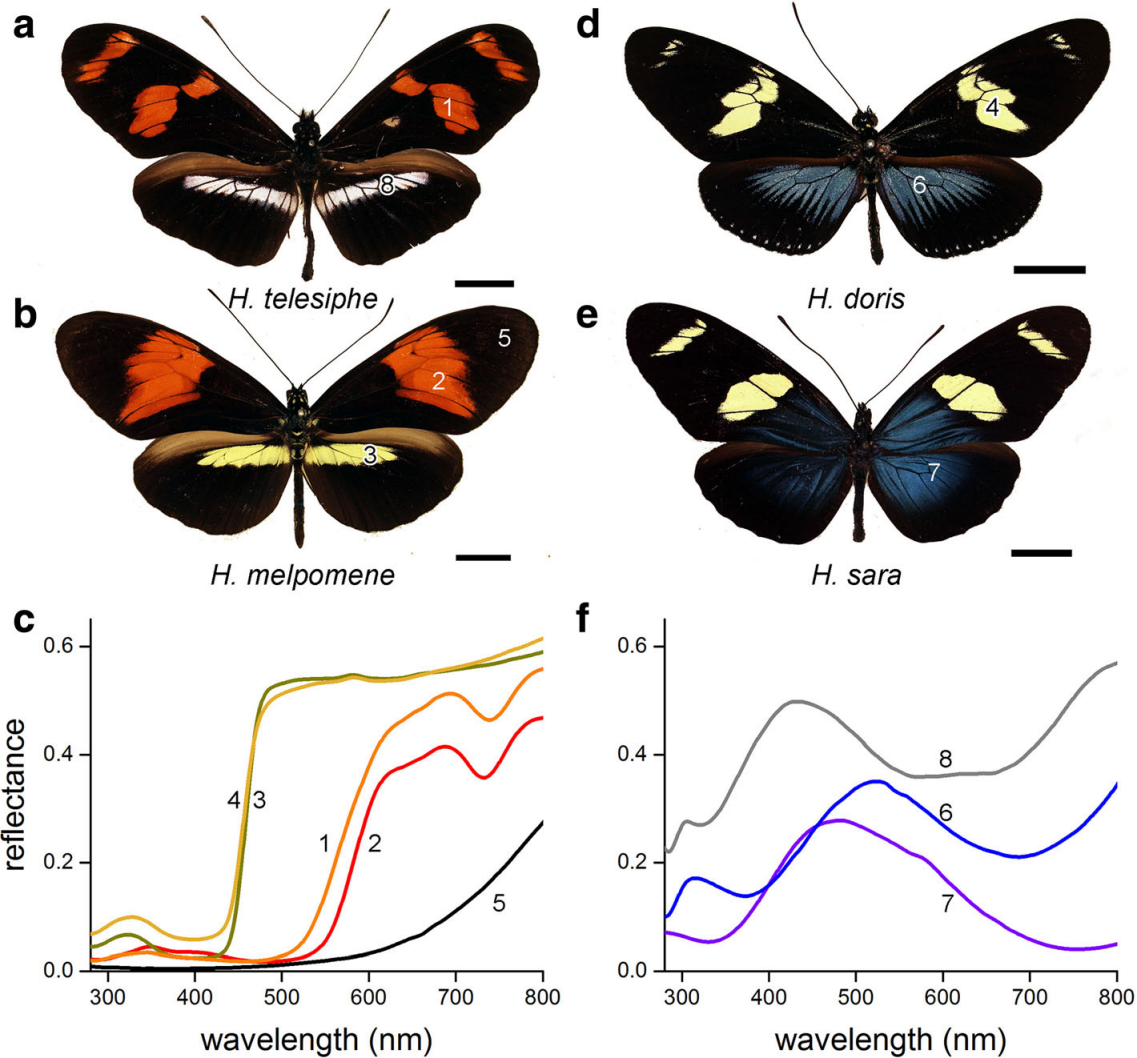
Representative coloration of *Heliconius* species. **a**, **b**, **d**, **e** Photographs of pinned specimen of *H. telesiphe*, *H. melpomene, H. doris*, and *H. sara,* respectively. Scale bars: 1 cm. **c**, **f** Reflectance spectra of the various colored dorsal wing areas measured with a bifurcated probe. The number near each spectrum corresponds to the wing location in panels **a**-**d** where the spectrum was measured

Figure 1 presents four *Heliconius* species from four of the seven major clades within the genus [24]. *H. telesiphe* and *H. melpomene* have dorsal forewings with prominent red wing patches, and dorsal hindwings with white (*H. telesiphe*) or yellow (*H. melpomene*) bands (Fig. 1a, b). *H. doris* and *H. sara* feature prominent yellow wing patches combined with bluish wing areas (Fig. 1d, e). To investigate the nature of the variously colored wing areas, we measured the reflectance spectra of these areas with a bifurcated optical probe (Fig. 1c, f). The reflectance spectra of the yellow- and red-colored areas (Fig. 1c, #1–4) are characteristic of pigmentary (or chemical) colored scales that absorb incident light in a restricted wavelength range [18, 29, 30]. The reflectance spectra of the velvet black wing areas (Fig. 1c, #5), found in all investigated heliconian butterflies, are typical for melanized wing scales [18]. As we will further discuss below, the spectra of the white and blue wing areas (Fig. 1f, 6–8) are characteristic of structural (or physical) colored scales.

### Spatial and spectral reflection properties of pigmentary-colored scales

**Fig. 2:**
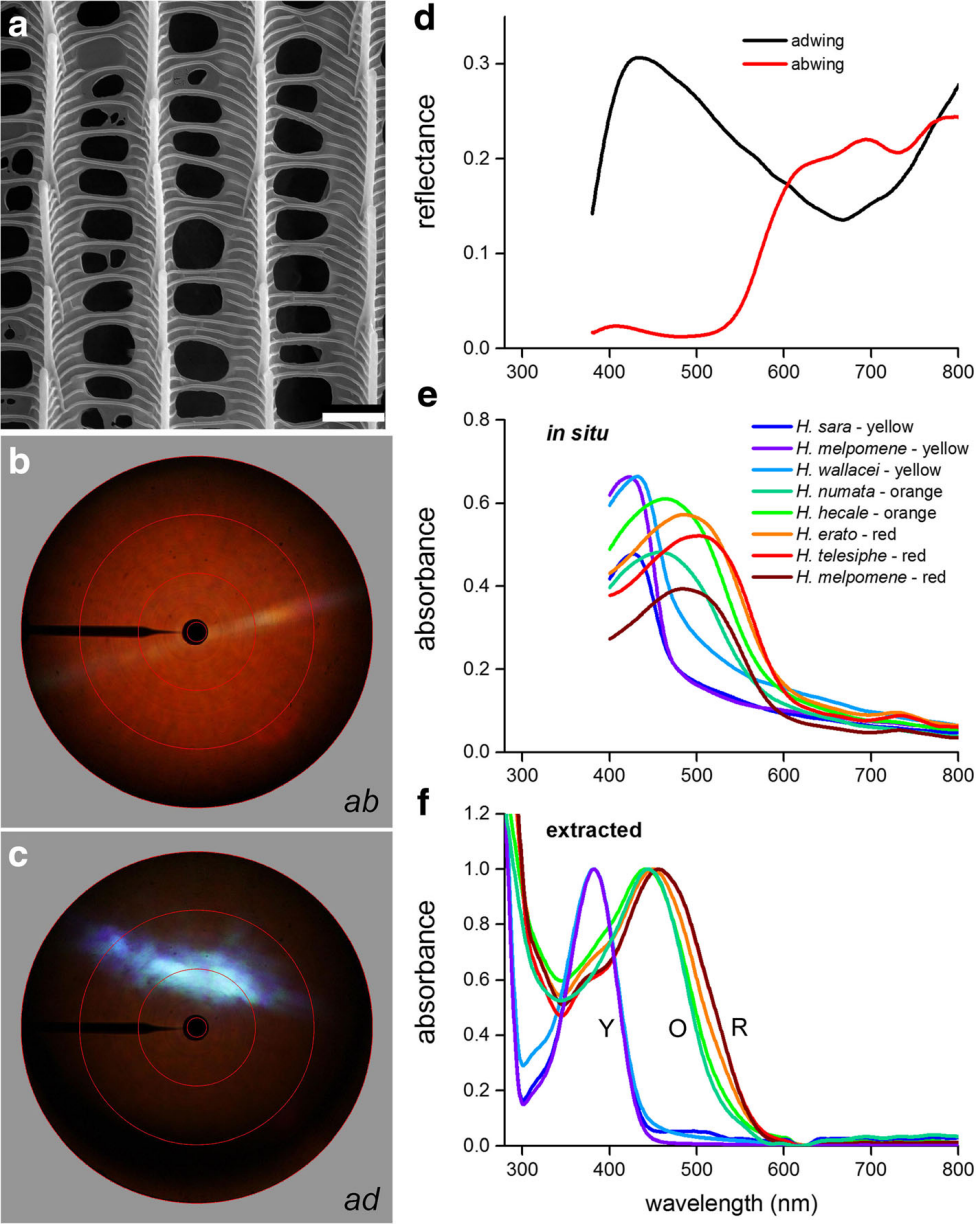
Ultrastructure, spatial scattering and spectral characteristics of pigmented wing scales. **a** Ultrastructure of a pigmented wing scale (scale bar: 2 μm). **b**, **c** Imaging scatterograms of a red-colored wing scale of *H. telesiphe* using local illumination on the abwing (upper) (**b**) and adwing (lower) (**c**) side. **d** Reflectance spectra of single red-colored wing scales of *H. telesiphe* measured from the adwing and abwing side. **e** Absorbance spectra of single wing scales immersed in refractive index matching fluid (*n* = 1.56). **f** UV-Vis absorbance spectra of extracted yellow (Y), orange (O) and red (R) pigments

In our analysis of the spatial and spectral reflection properties of the scales, we first focused on the pigmented wing scales. Figure 2a-c presents the case of a red wing scale of *H. telesiphe*. Scanning electron microscopy (SEM) confirmed that the scale has the classic nymphalid ultrastructure [18, 31, 32]. A set of parallel ridges, connected by crossribs, forming together the upper lamina, is mounted on an approximately flat lower lamina (Fig. 2a). Contrary to Gilbert et al. [32], we observe that all pigmented scales feature the same ultrastructure (see Additional file 1: Figure S1).

Figure 2b shows a scatterogram created by a narrow-aperture, white-light beam illuminating a small area of the upper lamina of a red scale. The scatterogram demonstrates that the red part of the white incident light is back-scattered into the full hemisphere (Fig. 2b). This is a characteristic feature of pigmentary-colored scales, where diffuse scattered light is spectrally-filtered by a short-wavelength-selective absorbing pigment [33]. The additional, weak-whitish line in the scatterogram, which is perpendicular to the ridges, shows that the set of parallel ridges acts as a diffraction grating (Fig. 2b); for similar cases see [18, 25, 30, 34].

Rotating the same scale by 180°, that is, illuminating the scale’s underside, yielded a scatterogram with a strongly-directional, bluish reflection and a much-reduced diffuse red scattering (Fig. 2c). We interpret this as that the lower lamina acts as an approximately flat, blue-reflecting thin film [18, 35]. Since the lower lamina’s reflection is low in the longer wavelength range, the red transmitted light will be scattered by the upper lamina, which thus contributes the relative weak, diffuse red scattering in Fig. 2c.

We note that the situation was fully opposite in the previous case of illumination, from the side of the upper lamina. Incident white light was then mainly scattered by the red-pigmented upper lamina, and the little short-wavelength light that reached (and was reflected by) the lower lamina, was again severely spectrally filtered by the upper lamina, resulting in a negligible contribution of short-wavelength light to the reflectance. To substantiate this interpretation, we measured the reflectance spectra of both sides of isolated, single scales with a microspectrophotometer (Fig. 2d). The reflectance of the abwing (upper) side of the red scale is very low at short wavelengths and high in the longer wavelength range, due to a pigment absorbing in the short-wavelength range. The reflectance of the adwing (lower) side has a major blue peak, indicating a thin film with thickness ~210 nm (see refs. [18, 35] for more details on how to extract the film thickness from reflectance measurements). Although for an ideal thin film with thickness 210 nm the reflectance should be zero at ~650 nm, the red-light-scattering upper lamina clearly causes a non-negligible contribution to the reflectance in the longer wavelength range. The spatial and spectral reflection properties of yellow scales were found to be basically similar as only the absorption is shifted to shorter wavelengths.

### Pigmentary basis of the coloration

To further characterize the pigments in the pigmentary-colored scales, we performed microspectrophotometry on single wing scales of *H. telesiphe* as well as of several other *Heliconius* species (at least 2–5 wing scales per specimen; Fig. 2e, Additional file 1: Table S1). To suppress the distortions by light scattering in the measurements of the absorbance spectra, we immersed the scales in a fluid with refractive index *n* = 1.56, closely matching that of cuticular chitin [27]. We found three pigments present in the scales: a yellow pigment with in situ peak absorption at ~420 nm, an orange pigment with peak absorption at ~480 nm, and a red pigment with peak absorption at ~520 nm (Fig. 2e).

We further studied the wing scale pigmentation by exposing the scales to a solution of 50:1 methanol: 1 M hydrochloric acid, which is known to dissolve ommochromes as well as their precursor 3-hydroxy-dl-kynurenine (3-OHK) [2]. The absorbance spectra of the obtained extractions, a yellow (Y), orange (O) and red (R) pigment (Fig. 2f), are highly similar to the in situ measurements (Fig. 2e), except for a distinct shift to shorter wavelengths of the extracted pigment spectra. This hypsochromic shift of ~20–45 nm must be attributed to the different chemical environment in the solvent [36, 37].

Most probably, the yellow (Y) pigment is 3-OHK [10, 38–41], while the orange (O) and red (R) pigments are the ommochromes xanthommatin and dihydroxanthommatin [2, 18, 32, 42, 43]. Previously, Bybee et al. [2] extracted the yellow pigment from the wings of 13 *Heliconius* species (eight of which overlap with the species in this study), and used mass spectrometry to demonstrate that the yellow pigment is indeed 3-OHK. We conclude that the dorsal yellow wing areas of the species depicted in Fig. 1 have scales with abundant 3-OHK, which causes the low reflectance in the (ultra)violet and the high reflectance at the longer wavelengths (Fig. 1e); the orange and red wing areas have scales that derive their color from (mixtures of) different ommochrome pigments [2, 18].

Curiously, the absorbance spectra of red wing scales feature a minor peak at ~720 nm (Fig. 2e), which is absent in the absorbance spectra of the extractions (Fig. 2f). The peak indicates trace amounts of (presumably) bile pigment, responsible for the quite noticeable dip in the reflectance spectra of Fig. 1e (#1,2) [44, 45].

### Spatial and spectral reflection properties of structural-colored scales

**Fig. 3:**
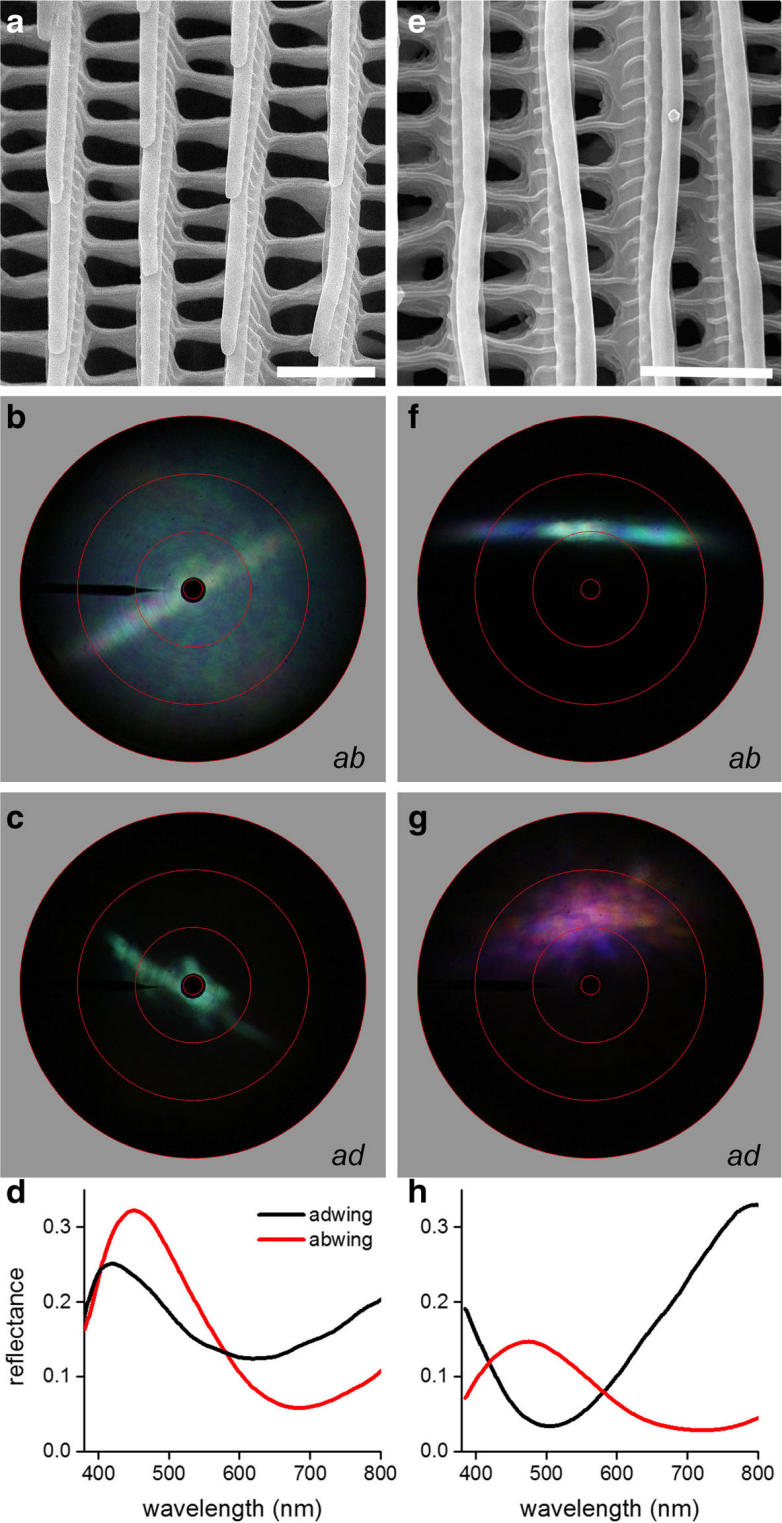
Optical characteristics of the structural colored scales of *H. telesiphe* (**a**-**d**) and *H. sara* (**e**-**h**). **a**, **e** Scanning electron micrographs of a scale from the white area of *H. telesiphe* and a scale from the blue wing area of *H. sara* (scale bars: 2 μm). **b**-**g** Imaging scatterograms of the abwing (upper) (**b**, **f**) and adwing (lower) (**c**, **g**) sides. **d**, **h** Reflectance spectra from both sides of the wing scale

Scanning electron microscopy of scales of the white and blue wing areas (Fig. 1a-d, #6–8) showed ultrastructures similar to that of the red scales (Figs. 2a and 3a, e). Interestingly, upon isolation of the scales of the white wing area of *H. telesiphe* it appeared that they were in fact blue, similar to those encountered in the closely related nymphaline butterflies [18]. Applying imaging scatterometry with the narrow-aperture light beam incident on the abwing (upper) side of a blue scale produced a diffuse, bluish scatterogram with a prominent line (Fig. 3b). Illuminating the adwing (lower) side of the scale yielded a scatterogram with a locally restricted, blue spot (Fig. 3c). The latter is due to the lower lamina acting as an approximately flat thin film, with thickness ~220 nm, as follows from the reflectance spectrum of the adwing side of the scale, measured with a microspectrophotometer (MSP) (Fig. 3d, c.f. Fig. 2d). The reflectance spectrum of the abwing side is very similar (Fig. 3d), meaning that the lower lamina of the thin film principally determines the scale color. The scales are unpigmented (not shown) and therefore most of the light flux entering from the normal, abwing side will pass the upper lamina and will then be partly reflected by the lower lamina. Most of that reflected light will pass the upper lamina again, where it will be diffused by the upper lamina structures, thus causing the bluish diffused scatterogram of Fig. 3b [18]; the line in Fig. 3b again results from the ridges acting as a grating.

Both cover and ground scales in the white band of Fig. 1a are blue, but as they form a stack of partly overlapping scales in situ, the multiple scattering causes an elevated reflectance spectrum with a hump in the blue wavelength range and thus a whitish color (Fig. 1a,f, #8). We encountered the identical situation in a number of nymphaline butterflies [18]. Interestingly, blue cover scales overlapping black ground scales cause the distinct blue wing areas of the dorsal hindwings of the Doris Longwing, subspecies *Heliconius doris doris* (Fig. 1d), again very similar as occurs in the nymphaline butterflies [18].

We initially expected that this would be also the case in the iridescent blue wing areas of the Sara Longwing, *H. sara* (Fig. 1e, #7). Scanning electron microscopy of the scales yielded in principle a similar scale structure as that of the blue scales of *H. telesiphe*, but closer inspection showed that the wing scale ridges are folded into a rather primitive multilayer of 2 to 3 layers (Fig. 3e). The scatterogram of the blue wing scales of *H. sara* illuminated from the abwing side is again line-shaped, similar to *H. telesiphe* due to diffraction by the ridges, but the wide-field, diffused blue scattering encountered in *H. telesiphe* is absent in the *H. sara* scale (Fig. 3f). The reason was revealed when inspecting the adwing side of the *H. sara* scale, which appeared to have a purplish instead of a blue color. The scatterogram of the adwing side is again spatially restricted (Fig. 3g), as in *H. telesiphe* (Fig. 3c), confirming that the lower lamina also here acts as a thin film reflector. However, the reflectance spectra measured from both scale sides with the microspectrophotometer (Fig. 3e) show that the physical mechanisms underlying the reflection properties of both sides of the *H. sara* scale must be quite different (Fig. 3h). The adwing reflectance spectrum indicates a thin film reflector of thickness ~180 nm in the lower lamina, causing a purplish instead of a bluish color. Furthermore, the blue *H. sara* scales appeared to contain melanin pigment (Additional file 1: Figure S2), which effectively suppresses incident light from the abwing side that is reflected by the lower lamina. We conclude that the abwing reflectance spectrum, peaking at ~500 nm, is due to the multilayered ridges, similar to the case of the well-known *Morpho* butterflies, where the intense blue coloration is caused by scales with multilayered ridges with up to 10 layers [25, 34, 46].

### Color tuning by pigment filtering

**Fig. 4:**
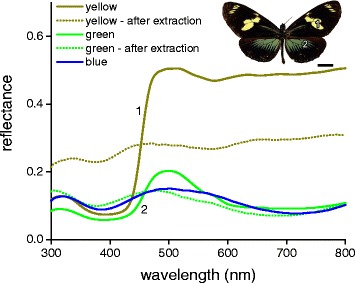
Spectral tuning in *H. doris*. The blue areas of the dorsal hindwings of the blue morph of *H. d. doris* (see Fig. 1d) are green in this specimen of *H. d. viridis* (inset; scale bar: 1 cm). The solid lines line show the reflectance spectra for the various morphs, while the dotted lines show the reflectance spectra measured after extraction of the pigment using acidified methanol (see Methods and Additional file 1: Fig. S2)

**Fig. 5:**
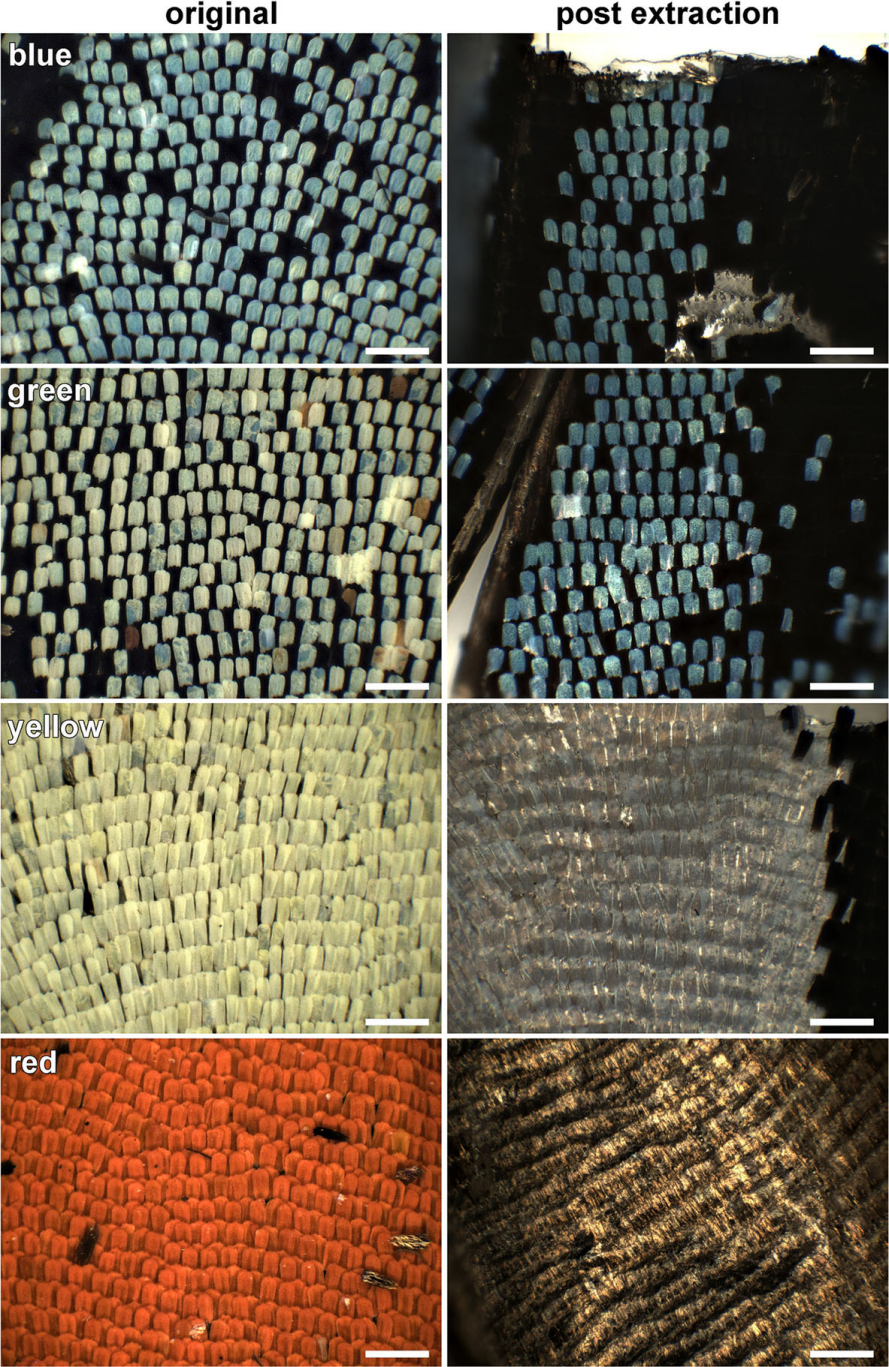
Pigment extraction. Wing scale lattice of different colored wing patches in *H. doris* morphs before (left) and after (right) extraction in acidified methanol (see Materials and Methods). While the blue patch remains blue colored, the green patch becomes blue and the yellow and red patches uncolored, confirming the presence of pigment in these scales. Scale bars: 200 μm

We encountered a modification of the simple blue scales of *H. telesiphe* in the Doris Longwing subspecies, *H. doris doris* and *H. doris viridis*. *Heliconius doris* is the only species exhibiting color polymorphism within populations [24, 47]. In the investigated butterflies, both subspecies have dorsal forewings with prominent yellow patches. The reflectance spectrum of the yellow wing area, measured with the bifurcated probe (Fig. 4, #1), demonstrates that the yellow color is determined by 3-OHK, which strongly absorbs scattered light in the short-wavelength range (Fig. 2f). The difference between the two *H. doris* subspecies is that the dorsal hindwings are blue in *H. d. doris* (Fig. 1d) and green in *H. d. viridis* (Fig. 4). As noted above, the blue scales of *H. d. doris* are unpigmented, but in the case of *H. d. viridis* the green scales clearly contain a substantial amount of 3-OHK. The reflected and scattered blue light, when spectrally filtered by 3-OHK, results in the greenish color (Fig. 4, #2). Indeed, after extraction of the 3-OHK pigment using acidified methanol (see above), the green-colored wing patch becomes blue (dotted line, Fig. 5).

### Phylogenetic relationships

We investigated the distribution of each of the seven types of colored scales identified in *Heliconius* in relation to the species’ phylogeny. The systematics of the genus *Heliconius* has been thoroughly studied due to their mimicry and rapid speciation [48–50]. We used the phylogeny of *Heliconius* proposed by Kozak et al. [24] to map the investigated pigmentary and structural traits. Additional file 1: Figure S4, S5 and Table S1 list six kinds of scales whose colors derive from either the presence of pigments (yellow, orange/red) or the scale (nano)structures (simple scales or multilayers in the ridges) for the investigated heliconians. Each trait represents a distinct scale type present on the wings of that species; species can have more than one type of colored scale. Previously, *Heliconius* scales have been classified according to ultrastructure and pigmentation as Type I (yellow or white), Type II (melanic) or Type III (orange or red) [32, 51–53]. Here we classify all three types as simple scales either with or without pigment, and to this classification we identify a new scale type with multilayered ridges.

All species possess melanin, which is therefore not included in either Additional file 1: Table S1 or Figure S4 and S5. Virtually all species have one or more of the three identified pigments in their brightly colored bars, with 3-OHK being the most abundant and its derivatives xanthommatin and/or dihydroxanthommatin being less frequent (Additional file 1: Figure S4A). Maximum likelihood ancestral state reconstruction suggests that scales with 3-OHK were present at the base of the genus *Heliconius*. Scales with xanthommatin and/or dihydroxanthommatin were also likely present at the base of both *Heliconius* and *Eueides,* although the data are less conclusive*.* The latter is likely an artefact of our sampling method, which sampled pigments from large colored wing patches due to the difficulty of pigment extractions from small wing patches. Several *Heliconius* species (*H. cydno, H. sapho, H. charithonia*) have minor red patches near the base of their hindwings that are too small for extraction. Thus dihydroxanthommatin is likely to be widely distributed across the genus.

The scale types whose colors derive solely from structural parameters (Additional file 1: Figure S4B and S5, right) are also highly variable among the various species and are not conserved within sub-clades, although no real trend in the data is apparent. Ancestral state reconstruction suggests a 50% likelihood that stacked simple-blue scales (white) as well as scales with multilayered ridge reflectors (blue) were present at the base of both the *Heliconius* and *Eueides* genera. Simple-blue scales covering black ground scales seem to be less common than the “white” scales, which result from stacking blue scales (Additional file 1: Figure S5, right). The green scales are the most interesting of all, as they appear only in *H. doris viridis,* which suggests that a new mechanism for uptaking 3-OHK in simple-blue scales must have evolved along the branch leading to *H. doris* (Additional file 1: Figure S5, right).

## Discussion

### The coloration toolkit of longwing butterflies

The wings of longwing butterflies have pigmentary as well as structural coloration. Pigments expressed in butterfly wing scales absorb scattered light in the short-wavelength range and thus typically act as long-pass spectral filters. We have identified three distinct absorption spectra associated with the pigments from colored wing patches (Fig. 2e, f): *i*) yellow 3-OHK, *ii*) orange xanthommatin and *iii*) red dihydroxanthommatin. 3-OHK is the precursor in the biosynthetic pathway by which xanthommatin and ultimately dihydroxanthommatin are synthesized within the developing scale cells [37, 41, 54]. While 3-OHK is mostly pure within the scales, it seems that the orange-red scale colors are finely tuned by mixing xanthommatin and dihydroxanthommatin (Fig. 2e). The absorption properties of the various pigments embedded in scales differ substantially from the pigments when extracted, probably because of the chemical environment of the scale [37]. The pigments produce the bright, localized color patches on *Heliconius* wings, and the velvet black frame of melanin-containing scales further increases the contrast of these patches (Fig. 1).

Structure-based colors are either due to the reflection of light at the scale’s lower lamina acting as a thin film (simple scales, e.g. in *H. telesiphe*, Fig. 3a-d) or at the ridge-reflector scales with multilayered ridges (e.g. in *H. sara*, Fig. 3e-h). When the simple scales are pigmented, incident light at the upper lamina will be scattered and spectrally filtered by the pigment present in the ridges (see above). The light fraction reaching the lower lamina will after reflection at the thin film be again spectrally filtered and hence contribute little to the overall scale reflectance. Consequently, the reflectance spectra of pigmented scales of the adwing or abwing side can be very different (Fig. 2d) [18]. Spectral filtering is absent in unpigmented scales where the lower lamina acts as a prominent thin film. The reflectance spectra measured with abwing (upper) and adwing (lower) illumination then are similar and feature typical thin film photonics [18, 55]. This is the case for the blue scales that determine the color in the blue and white wing patches (Fig. 3d).

On butterfly wings, scales are stacked like shingles on a roof so that a considerable fraction of the incident light passes the cover scales and reaches the underlying ground scales. These scales thus can also contribute to the wing reflectance. As the reflectance of the melanized black ground scales is negligible, blue scales overlapping black ground scales create a blue wing color as seen in *H. doris doris*, whereas blue scales stacked on top of each other on the wing substrate yield a desaturated white color as in *H. telesiphe* (Fig. 1a). The reflectance spectra of the white areas having peaks in the blue wavelength range (Fig. 1f, #8) reveal the presence of scales with blue thin-film reflectors.

Sweeney et al. [56] made the general statement, without further evidence, that thin-film interference creates the iridescent coloration of *Heliconius* butterflies, serving polarization signaling. However, here we have shown that the iridescent-blue wing scales of *H. sara* and *H. erato* are due to ridge-reflectors, as the scales have ridges folded into 2–3 overlapping layers (Fig. 3e) [57]. Together with the presence of melanin, light reflected from these scales is directionally scattered into a limited solid angle (Fig. 3f).

Structural colors can be enhanced or modified by pigments [14, 15, 58]. In the wing scales of *H. sara*, the underlying melanin pigmentation reduces scattering of transmitted (i.e. non-reflected) light that would otherwise desaturate the color signal. In the green-morph of *H. doris viridis*, pigmentary filtering of short wavelengths by 3-OHK occurs in blue-structural colored scales, such as those featured in the blue morph of *H. doris doris* (Figs. 4 and 5).

### Biological function of longwing coloration: Mimicry, speciation and sexual selection

Longwing butterflies benefit from mimicking other unpalatable butterfly species in their local habitat, as doing so spreads the cost of educating predators. The bright color patches of *Heliconius* wings serve as aposematic warnings to predators, advertising the distasteful nature of the butterflies [3]. The selective advantage of advertising distastefulness has led to a series of co-mimicry complexes in *Heliconius*, where multiple species converge on a similar color pattern [3, 59]. The most well-known system is that of *H. erato* and *H. melpomene* [60–62], which have similar wing patterns in local populations but diverse patterns between geographic regions. Indeed, the two subspecies of *H. erato* and the two subspecies of the highly color-polymorphic genus *H. doris* investigated here (see also Figs. 4, 5, Additional file 1: Figure S3-S5 and Table S1) share common structural motifs but have different pigments in their wing scales. It will be interesting to investigate coloration mechanisms in light of habitat-induced pressures. *Heliconius* species occupy light environments that can vary in brightness by one order of magnitude (see references in Seymoure et al. [63]), and can range from closed forest understory to bright open habitats. In order for the aposematism and mimicry to be successful, the longwing butterflies must continually fine-tune their colors to warn predators of their unpalatability. In this context, sexual selection is important for maintaining aposematism as it helps to select for specific shades of colors rather than the general appearance of the butterfly [4]. This has been recently demonstrated using behavioral tests where *H. erato,* given a choice of butterfly paper models that were identical in pattern, were shown to prefer 3-OHK yellow to a shade of yellow with a reflectance spectrum similar to that of co-mimics in the genus *Eueides.* By contrast, avian predators attacked butterfly paper models differing in shades of yellow at an equal rate under field conditions [64].

Sharing similar wing patterns brings about an additional selective pressure, namely that individuals must be able to differentiate conspecifics from heterospecifics in order to successfully reproduce. *Heliconius* butterflies have experienced a duplication of the UV-sensitive visual opsin pigment, which, along with the simultaneous expression of the UV-absorbing yellow pigment 3-OHK in the wings, has expanded the set of yellow colors that can in principle be distinguished by these butterflies [2, 10, 64]. Furthermore, the eyes of *H. erato* contain heterogeneously-expressed filtering pigments that enhance discrimination in the red wavelength range [65] via their filtering of a green-absorbing rhodopsin in some ommatidia, resulting in a red-sensitive receptor [66]. This is in contrast to butterflies like the lycaenid *Polyommatus icarus,* which uses a duplicated blue opsin to see green [67], or the papilionid *Papilio xuthus,* which uses both duplicated green opsins and filter pigments to produce red receptors [68].

The gene (*optix*) that globally regulates the development of red-pigmented *Drosophila* eyes has been re-deployed to control the expression of dihydroxanthommatin in the wings of *Heliconius* [23]. *Optix* is not, however, directly involved in regulating the expression of red filtering pigments in *Heliconius* eyes [69]. The evolutionary radiation of *Heliconius* butterflies is underlined by the diversification of pigments in the genus and the concurrent expansion of visual pigments that enhance the discrimination of similar colors [70].

Studies into the genetic processes determining coloration in *Heliconius* have indicated that surprisingly few genetic loci are involved in color pattern determination [23, 41, 62, 71]. Longwing species have undergone a remarkable adaptive radiation [41], driven in part by the color-pattern divergence between closely related species and mimicry-related color-pattern convergence between distantly related species [71]. A single gene controlling the expression of different wing patterns has also been found in *Papilio dardanus* [72, 73], which suggests that the genetic basis underlying diversity of wing patterns and species may be less complex than originally thought. It is truly remarkable that the observed vast diversity in species can be brought about by the convergence toward similar color patterns and relatively simple genetic mechanisms that control pigment expression together with a small number of optical mechanisms.

## Conclusion

In conclusion, we have unraveled the physical and chemical origins of the wing scale coloration in seven kinds of colored scales in longwing butterflies. Our results indicate that a limited set of pigments and structural mechanisms produce the diverse brightly colored wing patches observed in this group. Multiple mechanisms are often combined in non-trivial ways on both a species- and subspecies-specific basis to produce new or unique colors indicating high rates of ‘plasticity’ that might be driven by various biological pressures, e.g. mimicry and sexual selection.

## Additional file

Additional file 1: Figures S1-S6 and Table S1. (DOCX 2441 kb)
