# Supplementary material for: Longwing (Heliconius) butterflies combine a restricted set of pigmentary and structural coloration mechanisms
Source: BMC Evol Biol. 2017 Nov 21;17:226. doi: 10.1186/s12862-017-1073-1 (PMC5699198; doi:10.1186/s12862-017-1073-1)
Supplement: Additional file 1: — Figures S1-S6 and Table S1. (DOCX 2441 kb) [file 12862_2017_1073_MOESM1_ESM.docx]

# Supplementary Materials

for

**Longwing (*Heliconius*) butterflies combine a restricted set of pigmentary and structural coloration mechanisms**

by

Bodo D. Wilts*, Aidan J.M. Vey, Adriana D. Briscoe and Doekele G. Stavenga


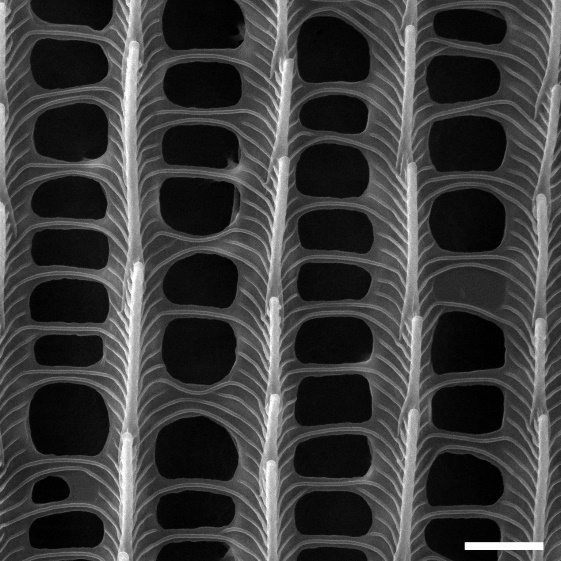


**Figure S1.** SEM of a yellow-pigmented pigmented wing scale of *H. sara* showing the same ultrastructure as the red-pigmented wing scale of *H. telesiphe* shown in Figure 2A.


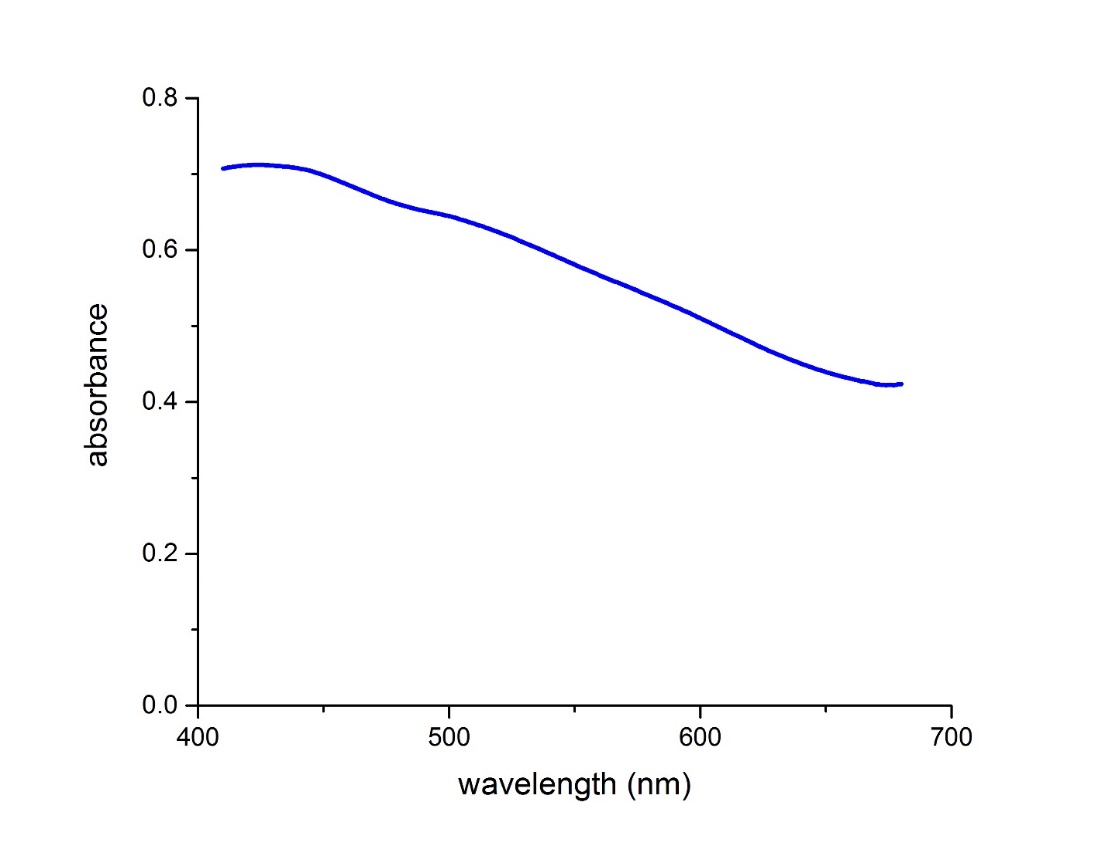


**Figure S2.** Absorbance spectrum of a single blue wing scale of *H. sara* immersed in refractive index-matching fluid.

**
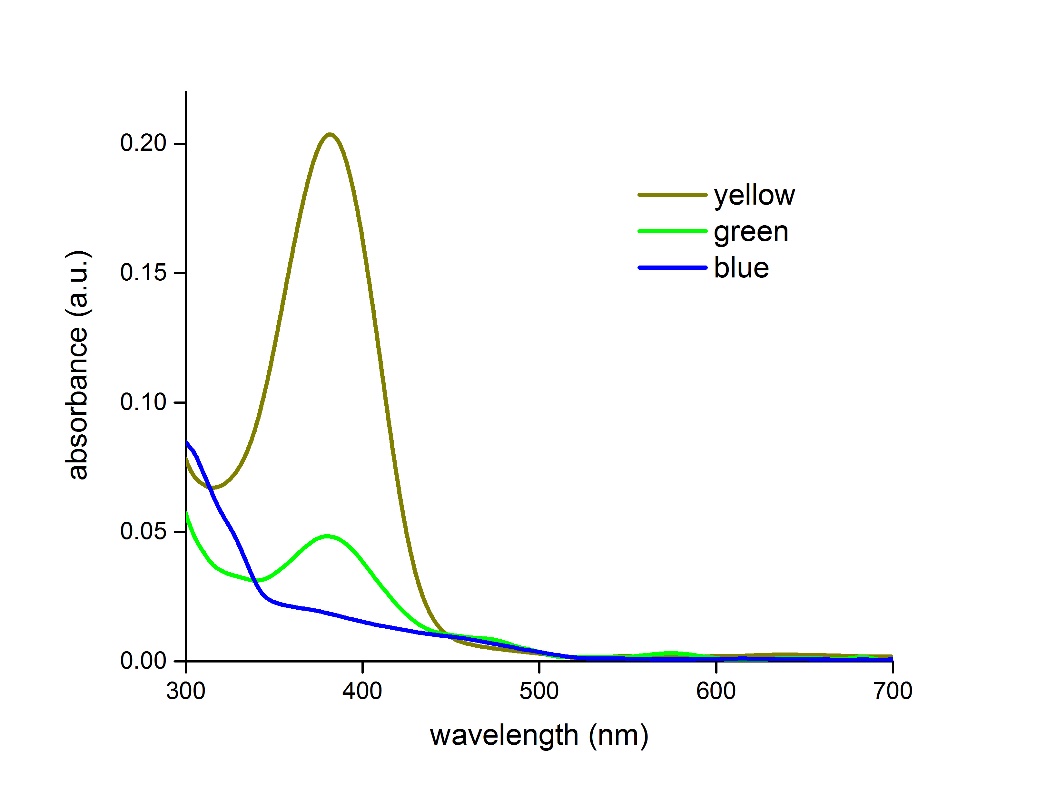
**

**Figure S3.** UV-Vis absorbance spectra of acidified methanol extracts from the different colored wing patches of the *H. doris* morphs (Fig. 5), confirming the presence of 3-OHK pigment in the green and yellow wing patches (c.f. Fig. 2F).

**
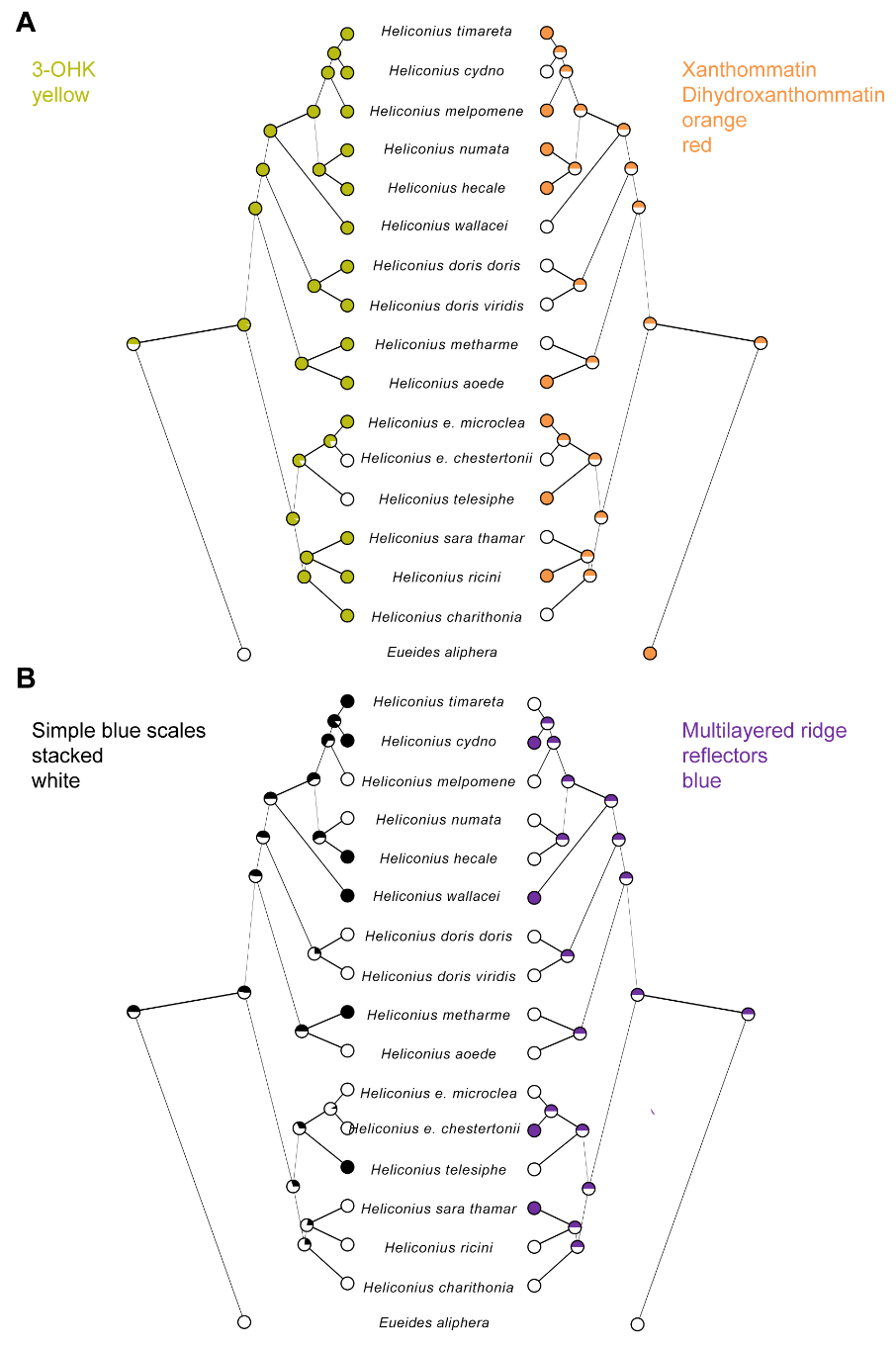
**

**Figure S4. Maximum likelihood reconstruction of yellow and orange pigmentary and white and blue structural scale coloration on the *Heliconius* phylogeny.**  Character mapping of four colored scale types found in *Heliconius* species and an outgroup in the genus *Eueides* on the topology of Kozak et al. [24] with branch lengths indicated. Filled circles indicate the presence of reflectance spectra of scales characteristic of: 3-OHK (yellow), xanthommatin/dihydroxanthommatin (orange), simple blue scales stacked (black) and multi-ridge reflectors coloration (blue). White indicates an absence of that scale type on the wings. Simple blue scales stacked produce scales that are white in color. Due to its universal presence, the presence of melanin has not been included. Circle graphs represent the likelihood that a particular trait was present in an ancestral node as estimated using Mesquite [28].


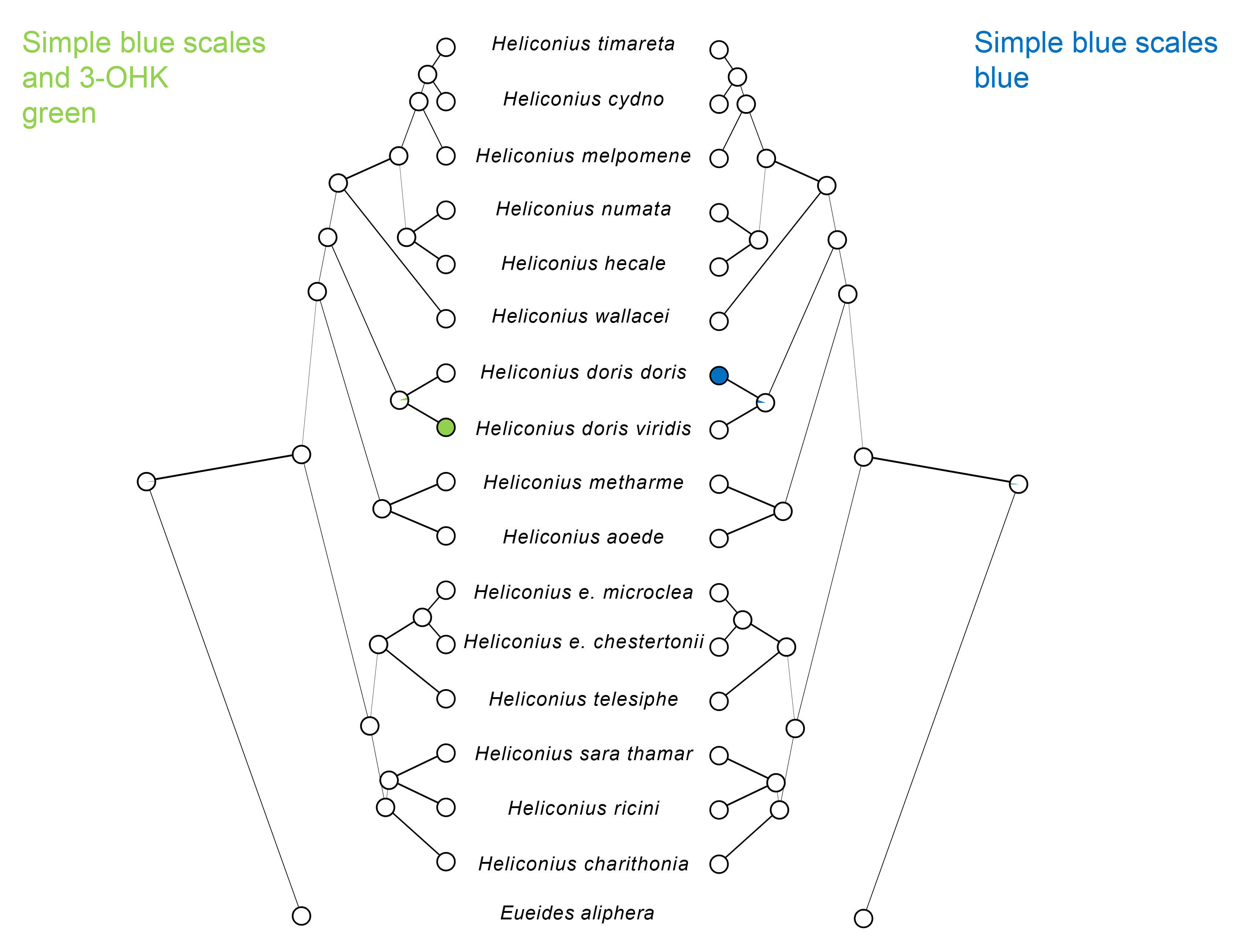


**Figure S5. Maximum likelihood reconstruction of red and green pigmentary and simple blue structural scale coloration on the *Heliconius* phylogeny.**  Character mapping of three colored scale types found in *Heliconius* species and an outgroup in the genus *Eueides* on the topology of Kozak et al. [24] with branch lengths indicated. Filled circles indicates the presence of scales with reflectance spectra characteristic of: simple blue scales filled with 3-OHK (green) or simple blue scales (blue). White indicates an absence of that scale type on the wings. Circle graphs represent the likelihood that a particular trait was present in an ancestral node as estimated using Mesquite [28].


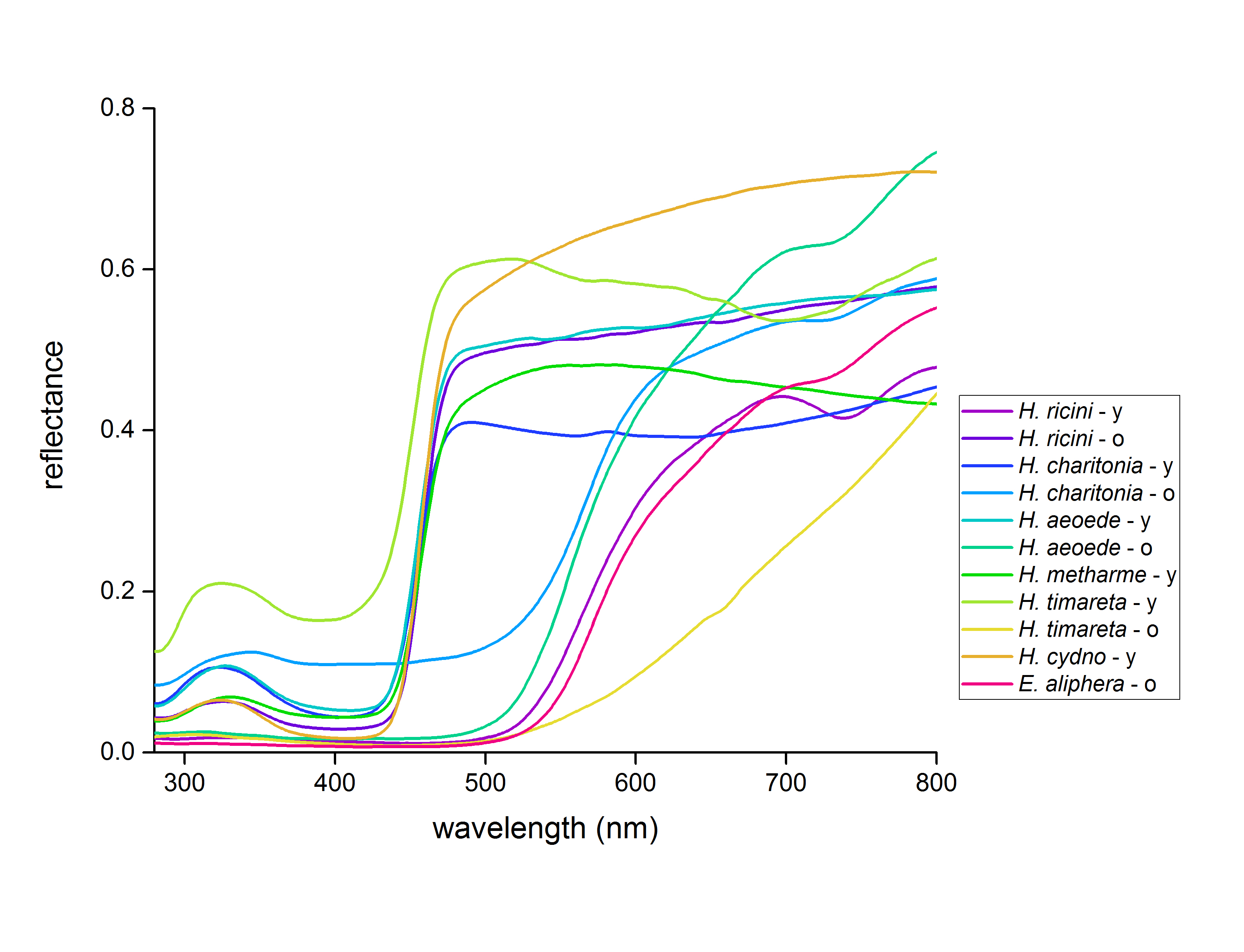


**Figure S6.** Reflectance spectra of the various colored dorsal wing areas measured with a bifurcated probe. This data was additionally used to assemble the dataset presented in Table S1 (c.f. Figs. 1 & 2).

**Table S1**. Overview of the pigmentary and structural coloration mechanisms encountered in the butterfly genus *Heliconius* as measured using reflectance spectroscopy.

|  | Pigment | | Structural | | | Both |
| --- | --- | --- | --- | --- | --- | --- |
| Species | Y | R/O | B | W | M | G |
| *erato* clade |  |  |  |  |  |  |
| *Heliconius telesiphe telesiphe^+^* |  | o |  | o |  |  |
| *Heliconius erato microclea^+^* |  | o |  |  |  |  |
| *Heliconius erato chestertonii^+^* | o |  |  |  | o |  |
|  |  |  |  |  |  |  |
| *sara/sapho* clade |  |  |  |  |  |  |
| *Heliconius charitonia^#^* | o | o |  |  |  |  |
| *Heliconius ricini^#^* | o | o |  |  |  |  |
| *Heliconius sara thamar^+^* | o |  |  |  | o |  |
|  |  |  |  |  |  |  |
| *aoede* clade |  |  |  |  |  |  |
| *Heliconius aeoede^#^* | o | o |  |  |  |  |
| *Heliconius metharme^#^* | o |  |  | o |  |  |
|  |  |  |  |  |  |  |
| *doris* clade |  |  |  |  |  |  |
| *Heliconius doris viridis* (green morph) *^*^* | o |  |  |  |  | o |
| *Heliconius doris doris* (blue morph)*^*^* | o |  | o |  |  |  |
|  |  |  |  |  |  |  |
| *wallacei* clade |  |  |  |  |  |  |
| *Heliconius wallacei flavescens^+^* | o |  |  | o | o |  |
|  |  |  |  |  |  |  |
| *melpomene* clade |  |  |  |  |  |  |
| *Heliconius melpomene^+^* | o | o |  |  |  |  |
| *Heliconius cydno^#^* | o |  |  | o | o |  |
| *Heliconius timareta^#^* | o | o |  | o |  |  |
|  |  |  |  |  |  |  |
| *silvaniform* clade |  |  |  |  |  |  |
| *Heliconius numata^+^* | o | o |  |  |  |  |
| *Heliconius hecale^+^* | o | o |  | o |  |  |
|  |  |  |  |  |  |  |
| *OUTGROUP SPECIES* |  |  |  |  |  |  |
| *Eueides aliphera^#^* |  | o |  |  |  |  |

*Pigment data shown in: ^+^Fig. 2 *Fig. 5 ^#^Fig. S6*

Abbreviations: Y: yellow, 3-OH-kynurenine
 R/O: orange/red, xanthommatin and/or dihydroxanthommatin
 B: simple blue scales
 W: simple blue scales stacked
 G: green, yellow-filtered simple blue scales
 M: multilayered ridge-reflector scales
